# Hormone therapy at early post-menopause increases cognitive control-related prefrontal activity

**DOI:** 10.1038/srep44917

**Published:** 2017-03-21

**Authors:** Romuald Girard, Elise Météreau, Julie Thomas, Michel Pugeat, Chen Qu, Jean-Claude Dreher

**Affiliations:** 1 Neuroeconomics, Reward and Decision-making Team, Institut des Sciences Cognitives Marc Jeannerod, Centre National de la Recherche Scientifique, 69675 Bron, France; 2 University Claude Bernard Lyon, Lyon 1, France; 3 Fédération d’Endocrinologie, Hospices Civils de Lyon et INSERM U1060 Institut CARMEN, Université Lyon 1, 69600 Oullins, France; 4 Psychology department, South China Normal University, Guangzhou, China

## Abstract

Clinical data have been equivocal and controversial as to the benefits to the brain and cognition of hormone therapy (HT) in postmenopausal women. Recent reevaluation of the role of estrogens proposed that HT may effectively prevent the deleterious effects of aging on cognition, and reduces the risks of dementia, including Alzheimer’s disease, if initiated early at the beginning of menopause. Yet, little is known about the effects of HT on brain activation related to cognitive control, the ability to make flexible decisions in relation to internal goals. Here, we used fMRI to directly test for a modulation of sequential 17β estradiol (2 mg/day) plus oral progesterone (100 mg/day) on task switching-related brain activity in women at early postmenopause. The results showed that HT enhanced dorsolateral prefrontal cortex recruitment during task switching. Between-subjects correlation analyses revealed that women who engaged more the dorsolateral prefrontal cortex showed higher task switching performance after HT administration. These results suggest that HT, when taken early at the beginning of postmenopause, may have beneficial effect on cognitive control prefrontal mechanisms. Together, these findings demonstrate that HT can prevent the appearance of reduced prefrontal cortex activity, a neurophysiological measure observed both in healthy aging and early dementia.

Fundamental behavioral and neurophysiological data in rodents and monkeys provide strong evidence for the existence of a window of opportunity allowing a neuroprotective effect of estradiol on the brain and beneficial effects on cognitive functions, when estrogens are initiated near the time of cessation of ovarian function, but not after a long period of ovarian hormone deprivation^1^,^2^. In humans, the effects of HT on brain and cognition are much more controversial. A number of studies reported that HT may prevent the deleterious effects of aging on cognition in early post-menopausal women, and reduces the risks of dementia, Alzheimer’s disease and mild cognitive impairment^3^,^4^, while HT administrated in postmenopausal women well beyond midlife does not improve cognitive skills^5^,^6^. Other studies have found that initiation of HT more than a few years after menopause is associated with an unchanged or increased risk of dementia and age-associated cognitive decline^5^,^6^. Moreover, Women’s Health Initiative Memory Study reported that postmenopausal HT with conjugated equine estrogens, when prescribed to women 65 years and older, produced deficits in cognitive functioning^7^,^8^.

The time between onset of menopause and when the treatment was initiated has been proposed to be a key factor explaining part of these discrepant observations^1^,^2^. According to the ‘critical time window’ hypothesis, HT effectively decreases cognitive decline in aging when it is initiated around the time of menopause, but not if it is administered decades later. Although there is a crucial need to test this hypothesis in women, the challenge is the difficulty of running a long clinical trial to test whether HT administrated early at the time of menopause effectively improves cognitive functions many years later.

Here, asking a different question, we aimed to investigate the impact of HT on a neurocognitive function known to be impaired later in life. Using a unique crossover placebo-controlled design, we carefully selected early postmenopausal women and investigated the effects of HT on brain activity related to cognitive control, a key function allowing flexible mental set-shifting, selection of actions and control of behavior in response to environmental context and internal goals. Since the magnitude of normal, age-related declines in cognitive control in women in their 50 s is very minimal, it has been a challenge to detect a behavioral index of estrogen benefits in early postmenopausal women^2^. In contrast, a neuroimaging approach focusing on brain regions subserving cognitive control has the potential to be sensitive enough to detect the effects of HT in the absence of behavioral change. Such experimental approach has previously proven useful to reveal estrogen effects on brain regions subserving verbal memory function, even in the absence of behavioral change^9^,^10^,^11^,^12^.

Here, we studied the effects of sequential 17β estradiol plus progesterone on brain activity engaged during task switching, by carefully selecting early postmenopausal women in a counterbalanced, randomized, crossover, double-blind and placebo-controlled design (Fig. 1, Table S1). Women took HT and a placebo during 8 weeks each. None of the women had ever taken HT before inclusion in the study. This unique crossover placebo-controlled design controlled for a number of confounding factors contributing to the discrepancies observed in the estrogen–cognition literature, including differences in the estrogen compounds administered, their route of administration, HT duration, age at HT initiation, inclusion of naive users HT^13^.

During fMRI acquisition, women performed a task switching paradigm twice (once under placebo, once under HT), which consisted in alternating between two letter discrimination tasks based on the color of the letters^14^ (Fig. 2A). Flexibly switching between cognitive operations is known to engage a bilateral prefronto-parietal network in healthy young individuals^14^,^15^,^16^,^17^ and older subjects have disruptively low activity in this network, particularly in prefrontal areas^18^. Based on these findings, we hypothesized that HT, as compared to placebo, will induce a relative increase in dorsolateral prefrontal activity during task switching in women at early post-menopause. Moreover, we tested whether this potential increased prefrontal cortex activity with HT plays a beneficial effect on task switching performance.

## Results

### Behavioral results during scanning

Mean responses times (RT) (Fig. 2C) and error rate (ER) (Fig. 2D) were assessed with a repeated ANOVA with condition (task switching, control) and treatment (HT, placebo) as factors. Subjects were slower (F_(1, 11)_ = 37.8, p < 0.001) and made more errors (F_(1, 11)_ = 11.6, p < 0.001) during task switching than during the control condition (average of tasks A and B performed separately), suggesting that the switching condition engaged additional cognitive processes compared to those needed for the control condition. There was no main effect of treatment on RT (F_(1,11)_ = 0.4, p = 0.5) or error rates (F_(1,11)_ = 0.6, p = 0.4), nor interaction between conditions and treatment types (RT: F_(1,11)_ = 0.7, p = 0.4;error rate: F_(1,11)_ = 0.002, p = 0.96). Another ANOVA including treatment (HT *versus* Placebo) and type of trials (switch *versus* repeat) was performed separately for each task switching condition. This analysis revealed a task switch cost on RT (F_(1, 11)_ = 18.8, p < 0.001) and error rates (F_(1, 11)_ = 7.9, p < 0.01) (i.e. higher RT and error rates for switch compared to repeat trials), and no main effect of treatment on RT (F_(1, 11) = _0.0035, p = 0.95) and error rates (F_(1, 11)_ = 0.3, p = 0.5). The interaction between switch cost and treatment was not significant (RT: F_(1, 11)_ = 0.001, p = 0.97; error rate: F_(1, 11)_ = 2.2, p = 0.1). The absence of treatment effect on reaction time or error rate is in line with findings from the large KEEPS trial^19^, which reported no cognitive benefit of estrogens in early postmenopausal women.

### Estradiol levels

The day of inclusion in the study (i.e. before HT or placebo treatment), plasma estradiol level was 29.9 ± 23.7 pmol/L, confirming women’s perimenopausal status. This baseline estradiol level was significantly lower than when women were under HT (142.7 ± 70 pmol/L) (F_(1, 11)_ = 34.6, p < 0.001) and did not differ significantly from when they were under placebo (20.6 ± 2.9 pmol/L) (F_(1, 11)_ = 2.12, p = 0.17). When directly comparing estradiol concentrations on the two scanning days, HT increased plasma estradiol level relative to placebo (F_(1, 11)_ = 34.6, p < 0.001) (Fig. 2B).

### fMRI results

#### Brain regions engaged by task switching compare to control, regardless of treatment type

We first searched for brain regions engaged by “task switching” relative to the control condition (Fig. 2, Table 1). Increased BOLD response was mainly observed in a bilateral fronto-parietal network including the bilateral dorsolateral prefrontal cortex (DLPFC), anterior lateral PFC (i.e., aLPFC), the anterior cingulate cortex and the inferior lateral prefrontal cortex. The bilateral inferior (iPC) and superior (sPC) parietal cortex, the anterior insula (BA 47) and cerebellar regions also showed higher activity during task switching relative to control. The engagement of this bilateral prefronto-parietal network is consistent with previous fMRI studies assessing task switching using the same paradigm in healthy subjects^14^,^15^,^16^,^17^,^18^. Conversely, relative deactivation in a large anterior medial frontal cortex region was observed in the opposite comparison (control >task switching), consistent with the default mode network (Fig. S1A).

#### Direct comparison between HT and placebo during task switching

When directly comparing HT administration relative to placebo (p < 0.001, uncorrected), women showed increased BOLD signal during task switching relative to control in a bilateral frontal network including the right DLPFC (BA 46), the ventro-lateral prefrontal cortex and the anterior cingulate cortex (BA 24) (all: p < 0.05, SVC FWE corrected). The inferior frontal junction/anterior insula bilaterally (right: p < 0.05, FWE cluster-wise corrected) showed an increased activity, as well (Fig. 4, Table 2). Interestingly, none of the parietal regions engaged in the main effect of task reached statistical significance in this direct comparison (p > 0.05).

Direct statistical comparison between treatment types showed that placebo, relative to HT (p < 0.001, uncorrected) elicited higher activity in the anterior medial frontal cortex, middle and posterior cingulate gyrus (p < 0.05, FWE cluster-wise corrected), striatum, bilateral superior and inferior parietal cortex (p < 0.05, FWE cluster-wise corrected), temporal cortex and occipital regions (p < 0.05, FWE cluster-wise corrected) (Fig. S1B, Table S2).

#### Correlation between brain activity and behavioral performance

To restrict our analysis to the brain regions showing higher BOLD response under HT in the task switching condition relative to control (Fig. 4A), we created an inclusive binary mask (p < 0.05, uncorrected). We then applied it to the brain regions showing a positive correlation between BOLD activity and the percent correct performance in task switching under HT (p < 0.05, uncorrected). The betas from the activated voxels under HT were extracted at the peak-voxel in the DLPFC (x = 39, y = 5, z = 64 and x = −39, y = 8, z = 61) (Fig. 4C) and in the ACC (x = 3, y = 26, z = 22) (p < 0.05, uncorrected) to plot the correlation. Moreover, direct comparison between correlation coefficients from the two conditions showed that women who engaged more the left and right DLPFC had better performance in the task switching condition after HT administration than after placebo (Fisher z-transformation, Z = 1.95, p < 0.05). No negative correlation between percent correct performance in task switching and the brain network engaged under HT relative to control (p > 0.05).

## Discussion

The beginning of post-menopause provides a unique model to study how naturally low endogenous baseline estrogen levels influence cognitive control-related brain activity. The key question addressed by the current study was to investigate the modulatory effect of HT on early post-menopausal women’s brain activity while engaged in a cognitive control paradigm. The ability to dynamically switch between tasks is a critical function allowing flexible behavior and seemless transitions between cognitive operations. It has been difficult to distinguish the effects of aging and changes in gonadal steroid hormones levels on brain activity because endocrine and neural senescence overlap in time and are mechanistically intertwined in complex feedback loops. Our careful selection of women in a specific age range (around 52 years old) allowed us to pinpoint the specific effect of HT on brain activity during task switching. Our neuroimaging findings indicate an increased lateral prefrontal activity associated with HT administration, relative to placebo (Fig. 4A and B). A number of neuroimaging studies investigating cognitive control and working memory functions have demonstrated reduced or abnormal pattern of prefrontal activity in healthy older subjects^20^,^21^ and in early dementia^22^. Our results showed that among postmenopausal women taking HT, those increasing the most activity of the dorsolateral prefrontal cortex were also the ones who performed better in task switching (Fig. 4C). The stronger between-subject positive correlation between dorsolateral prefrontal activation and percent correct task switching performance observed with HT compared to placebo, indicates that such increased BOLD signal is beneficial for cognitive performance.

Our current crossover placebo-controlled design has a number of advantages because it is controlled for a number of factors contributing to the discrepancies in the estrogen–cognition literature. These factors include differences in the estrogen compounds used, their route of administration, HT duration, age at HT initiation, inclusion of both past and current users HT and cyclic *versus* continuous regimens^13^. In contrast, in our study, none of the women had taken HT before and each crossover woman serves as her own control using the exact same dose, duration and mode of administration of HT.

There has been a lack of human neuroimaging studies investigating the neurofunctional influence of HT on cognitive control in early post-menopausal women and, to the best of our knowledge, our study is the first to investigate the impact of estrogen plus progesterone on brain activity during a task switching paradigm. Our findings extend to the cognitive control domain a number of neuroimaging results obtained in postmenopausal women using estradiol alone and investigating working memory functions^11^,^12^ or retrieval of words from memory^23^,^24^, indicating that estrogen enhances prefrontal functions. Consistent with this effect, studies combining functional neuroimaging and pharmacological manipulation inducing ovarian hormone suppression in healthy young women demonstrated decreased activation in prefrontal cortex, anterior cingulate and medial frontal gyrus during verbal encoding^9^ and the Wisconsin Card Sorting test^25^. These effects were reversed when estradiol levels returned to normal levels^10^,^25^. Consistent with these neuroimaging studies, preclinical data also attest to a neuroregulatory role of estradiol on the prefrontal cortex. In female monkeys, estrogen increases spine density and plasticity in the PFC^26^ while ovariectomy decreases spine density in the PFC of female rats^27^. Potential neurophysiological bases of this effect are that the PFC is one of the highest estrogen binding sites in the female brain, with estradiol PFC concentrations being approximately twice higher compared with the temporal lobe and seven times higher compared with the hippocampus^28^.

Our fMRI results indicate that neuroimaging is efficient to detect effects of HT not yet visible with behavioral measures^2^. Indeed, the neurofunctional differences we observed between HT and placebo were evident despite a lack of direct behavioral differences in task switching performance between HT and placebo, indicating that fMRI can detect effects of sequential estrogen plus progesterone on prefrontal cortex not yet apparent with behavioral measures. This absence of task switching differences between HT and placebo at the behavioral level is consistent with the absence of substantial effect of HT on executive function or episodic memory in recently menopausal women^2^,^29^,^30^. These clinical trials in midlife women are also in agreement with results from larger trials in older women^2^,^31^. However, reports from the longitudinal Study of Women’s Health Across the Nation suggest that natural menopause has a slight effect on some aspects of cognitive function that may be time limited. That is, women who initiated HT after enrollment but before their final menstrual period and then discontinued the HT had a beneficial cognitive effect, whereas women who initiated HT after the final menstrual period had a detrimental effect on cognitive performance^32^.

A number of neuroimaging data demonstrated neuroregulatory effects of sex steroid hormones on working memory or verbal episodic memory functions in women. However, these effects were, by definition indirect and have largely been inferred from changes occurring during the normal menstrual cycle, after the administration of ovarian hormones to surgically menopausal women or in pharmacological models of menopause^10^,^25^,^33^. In contrast, the current results directly indicate that in women with naturally low estradiol level, early HT initiation increases lateral PFC cognitive control-related activity.

The absence of behavioral effect of HT in the relatively young women (around 50 y.o) we tested was expected from the literature. Since the magnitude of normal, age-related declines in cognitive control in women in their 50 s is very minimal, it has been a challenge to detect a direct behavioral index of estrogen benefits in early postmenopausal women^2^. This is not only true in the domain of cognitive control, but also in the domain of working memory, which engages the same brain circuitry (dorsolateral prefrontal and intra-parietal region). Moreover, practice effects are likely to mask more subtle effect of HT on cognitive control function. Subjects followed a training session on the day before each scan to minimize performance differences across HT and placebo conditions. Thus, our use of overlearned cognitive material likely limited our ability to detect performance changes due to HT because of ceiling effects. Not only the behavioral performance did not change on average with hormonal state, but also the variance of these measures was likewise similar across the HT and placebo conditions. Thus, on the basis of the present data, we cannot rule out the possibility that cognitive impairments with placebo would have been seen with other cognitive tasks if they had not been overlearned.

Note, that it can in fact be considered an advantage to observe no change in task switching performance between HT and placebo in women in their 50’s. Indeed, if women had shown worst performance in task switching either under placebo or under HT while engaging more robustly the same fronto-parietal network with HT, this would have obscured the neuroimaging interpretation. Similar problems of interpretation in neuroimaging occur for example when comparing healthy subjects with patients with schizophrenia, who generally perform more poorly than healthy controls in terms of working memory^20^. In such situation, it is difficult to interpret the changes in brain activation between patients and controls as being truly due to the disease rather than to the change in performance. Similarly, in the current study, if we had observed differences in task switching performance between HT and placebo, it would have been difficult to know whether between groups differences in brain activation would have been due to HT alone or to the change in behavioral performance.

Studying the impact of HT on the neural substrates of cognitive control in early post-menopausal women has important implications for health and neurological diseases because decline in executive functions are central to theories of normal age-related changes in cognition^20^,^34^ and because of potential benefits of HT on reducing the risks of neurodegenerative disorders, known to disrupt prefrontal executive functions^22^,^35^. Executive functions deficits are frequent in neurodegenerative disorders such as Alzheimer’s disease, frontotemporal degeneration, and its subtype frontotemporal dementia^36^,^37^,^38^. Executive deficits may also predict conversion from mild cognitive impairment to Alzheimer’s disease^39^,^40^,^41^ and the progression of FTD^42^,^43^. Recent cross-sectional studies have reported a relative increased activity during task performance in prefrontal regions in older subjects or patients with neurodegenerative diseases, including frontotemporal dementia or Alzheimer’s disease, when comparing to younger or healthy age-controlled subjects^44^,^45^,^46^,^47^. However, this increased pattern activity was not observed over time in longitudinal studies and may reflect compensatory effect to maintain cognitive function^45^. Since cognitive flexibility is an outcome variable which declines with age and predicts dementia later in life^37^,^38^,^39^,^40^,^41^,^43^, our results suggest a beneficial effect of HT on cognitive control brain function if HT starts early at the beginning of post-menopause.

Our results demonstrate short-term effects of HT on brain activity during task switching. Several caveats need to be mentioned. First, the sample size is relatively low for a fMRI study. However, given the inclusion criteria (recruitment of healthy hormone therapy naive early postmenopause women) were very stringent and the repeated measures design required (each woman had to be scanned twice), this is understantable. In fact, such sample size conforms to the literature on hormonal effect in women^48^,^49^,^50^,^51^. Second, the long-term effects of the same intervention, repeated month after month, may or may not be similar throughout the early postmenopause and into the late postmenopause. Third, effects may apply to healthy postmenopausal women but not necessarily to postmenopausal women with neurodegenerative disorders. Finally, the effects observed on brain activity may or may not be associated with effects that are clinically observable.

Our study, combining pharmacological manipulation, endocrinology and functional brain imaging in healthy women, helps to specify the effect of hormonal modulation on brain activity. Our results have implications for understanding brain responses benefits of sequential estradiol plus progesterone therapy in midlife women. Together, our study provides evidence of a neurofunctional modulation of cognitive control mechanisms by HT and establishes a neurobiological foundation for understanding the beneficial impact of gonadal steroid hormones on the brain regions engaged in task switching functions.

## Methods

### Subjects

Fifteen healthy, right-handed, non-smoking women in stage + 1a of early postmenopause (according to STRAW + 10 terminology)^52^ were carefully selected through advertisement in local newspapers. Three of them were excluded from the analyses because of problems encountered during scanning. The mean age of the twelve remaining women was 51.8 ± 2.1 years old (range 48–55 years old). Women were all at the end of their menopause transition (9.6 ± 5.7 [range = 6–24] months of amenorrhea after the last menstrual period) at the time of their inclusion. By definition, menopause transition ends when a woman has gone 12 months without having her period. We chose to initiate HT early in this group of women (the time between the last menses and HT initiation was less than a year) to maximally take advantage of a possible beneficial effect of HT on cognitive control. None of the women had ever taken hormonal therapy before inclusion in the study. All women were french native speakers with at least a high school level of education. Women were initially screened by phone interview, and then underwent a full clinical interview and a physical exam (performed by M.P). Exclusion criteria included any past or present use of estrogen or progestin, current or previous mental disorders, depressive tendency assessed by the Beck Depression Inventory (DBI), pathological gambling assessed by the South Oaks Gambling Screen (SOGS), hypertension, diabetes, elevated cholesterol, breast cancer and MRI contraindications. The study was approved by the local ethics committee (Centre Léon Bérard, Lyon, France) and written informed consent was obtained from all subjects in accordance with the principles of the Declaration of Helsinki.

### Hormone and Placebo treatment

**Figure 1:**
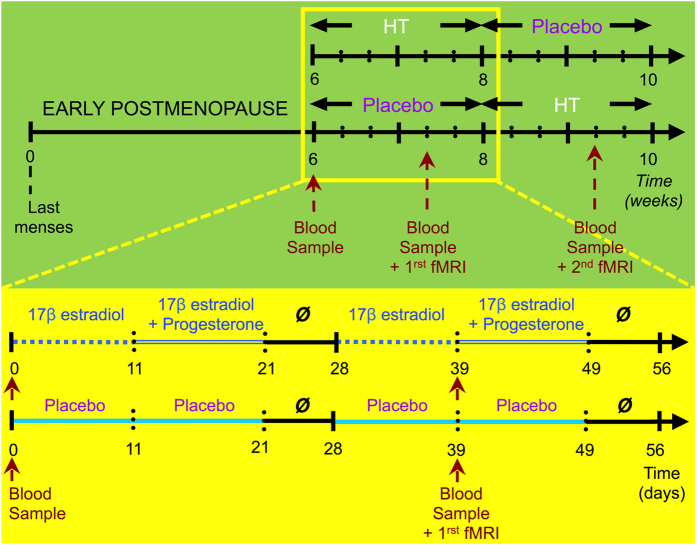
Experimental design. Early postmenopausal women were enrolled in a double blind, randomized, placebo-controlled crossover study. They took a daily pill of either HT (respectively placebo) for two cycles of 28 days each, followed by two consecutive months of placebo (respectively HT). For the first 11 days of a ‘restored’ menstrual cycle (under HT), pills contained 17β estradiol (2 mg/day). From the 12^th^ to the 21^th^ day, the pills contained an addition of 100 mg/day of progesterone. This was followed by a week washout period. This cycle was repeated for a second month (see yellow rectangle). After these two months, a new cycle started: women receiving HT first were administrated with a placebo containing 2 mg/day of inactive substance for the first 11 days and 102 mg/day for the following 10 days. This cycle was repeated during a last cycle of 28 days. The order of receiving HT and placebo was randomly assigned and counter-balanced. Blood samples were collected at the beginning of the study and on each day of scanning (once under HT and once under Placebo).

**Figure 2:**
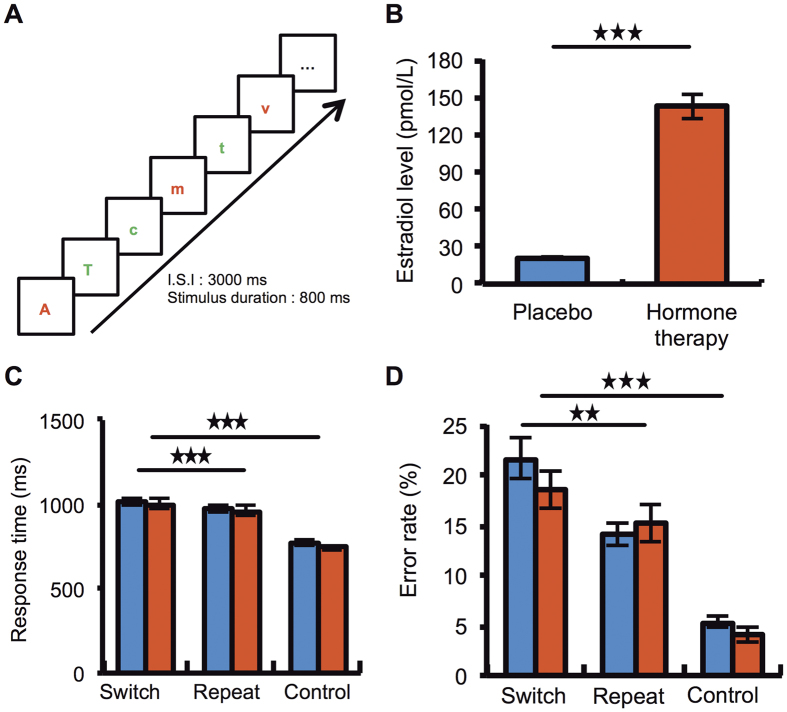
Tasks and behavioral results. (**A**) Women responded to visually presented letters by pressing response buttons with their right or left hand. Each condition was cued by a distinct written instruction displayed at the beginning of the run. In *task-switching condition*, subjects had to switch between two letter-discrimination tasks depending upon the color of the letter. If the letter was red, subjects performed a *vowel–consonant discrimination task* (vowel: right button; consonant: left button). If the letter was green, subjects performed a *case discrimination task* (upper case: right; lower case: left). In two conditions used for control, women performed each of these two vowel/consonant and upper/lower case discrimination tasks in separate blocks of trials. The control was the average of these two simple discrimination tasks. Stimuli appeared with fixed timing (inter stimuli interval = 3 s, stimulus duration = 800 ms) in a pseudo-randomized order. **(B)** *Estradiol measures*. Estradiol level (pmol/L) measured on the two days of the scanning sessions under HT and Placebo. **(C)** *Mean and SEM response time*. During the task switching condition (i.e. both switch and repeat trials from the task switching condition), subjects were significantly slower than in the control condition (p < 0.001). A switch cost was also observed when comparing switch trials to repeat trials (p < 0.001). **(D)** *Mean and SEM Error rate*. Z error cost was observed in women regardless of treatment type, that is, women made more errors in switch compared to repeat trials (p < 0.01) and control (p < 0.001) trials.

Our choice of the hormonal treatment (sequential estrogen-plus-progestin) was done to reproduce the hormonal environment of physiological early postmenopause^53^,^54^ and also on the fact that unopposed estrogen (the supplementation of endogenous estrogens without a progestagen) can result in endometrial hyperplasia, a precursor to endometrial cancer. Each woman received HT and a placebo in a random and counter-balanced order (half of women started the experiment with 2 months of HT while the other half started it after taking a placebo for 2 months) (Fig. 1, Table S1). For the first 11 days of a ‘restored’ menstrual cycle, women took a daily pill containing 17β estradiol (2 mg/day). From the 12^th^ to the 21^st^ day, women took 100 mg/day of oral progesterone in addition to estradiol. This was followed by a week washout period. This same cycle was repeated during a second month. The group of women receiving HT during the first 2 months of the experiment took placebo pills on week 8 for 11 days and for the following 10 days. After another week washout period, the placebo cycle was repeated. The visual aspect and dose of the placebo pills was exactly the same as those with active treatment. None of the women included in the analysis experienced menstrual cycle recovery after each cycle of estradiol and progesterone therapy. All pills were manufactured by the Pharmacy Division of the ‘Groupement Hospitalier Est’ (Bron, France). Women were asked to take their pills every day at the same time. On the scanning days, women took their pills one hour before the session.

### Stimuli and Task

**Figure 3:**
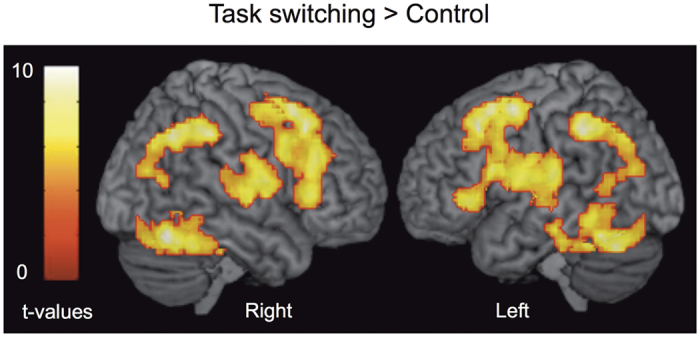
Brain regions engaged by task switching compare to control, regardless of treatment type. Data for brain regions commonly activated by task switching relative to control, regardless of hormonal status, were overlaid onto a 3D-rendered brain (Display threshold: voxel-wise p < 0.001, uncorrected; cluster-wise p < 0.05, FWE corrected).

**Figure 4:**
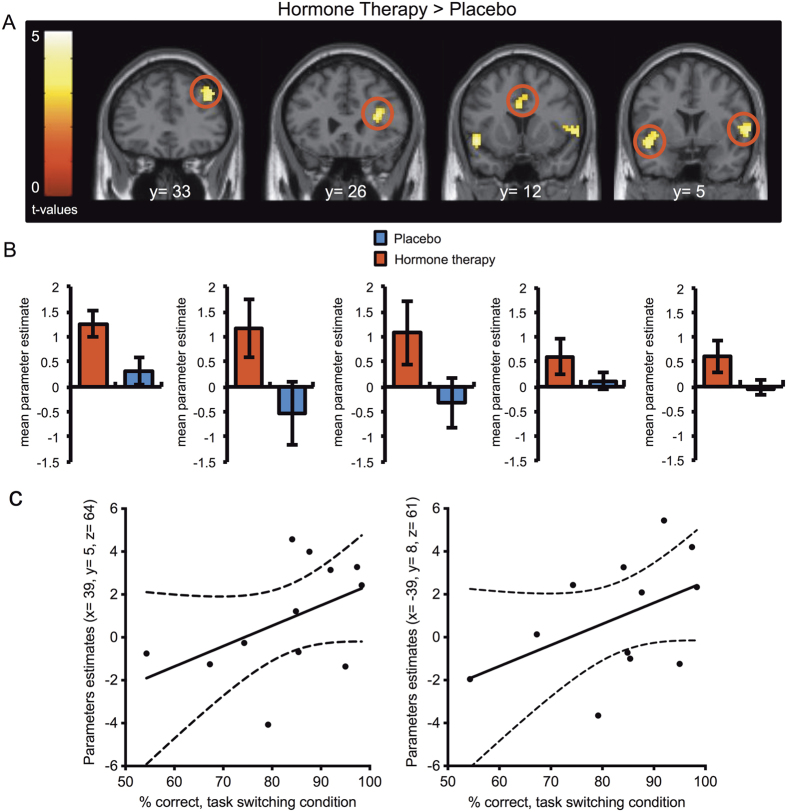
Direct comparison between HT and placebo during task switching relative to control. (**A**) Significantly greater activation after HT administration compared with placebo during task switching blocks relative to control (Display threshold, p < 0.005, uncorrected). The activation maps are overlaid on coronal sections of a structural template MRI. The color bars represent *t* values. (**B**) Mean and SEM of the contrast values in functional clusters of interest during task switching condition when comparing HT and placebo. From left to right, in the right dorsolateral prefrontal cortex, the right ventro-lateral prefrontal cortex, the anterior cingulate cortex and the inferior frontal junction/anterior insula. (**C**) Positive correlation between % correct performance and parameter estimates in right and left DLPFC in the task switching > control conditions in women under HT.

On each scanning day, women had to respond to visual stimuli consisting of single alphabetical red or green letters (vowels or consonants, lower or upper case) presented at the center of the screen, by pressing on one of two response buttons held in each hand (Fig. 3A). Each fMRI session was composed of 5 runs with 3 conditions (24 trials in each condition). A cue displaying a distinct written instruction was provided at the beginning of each condition. In the *vowel-consonant discrimination* condition (task A), subject had to press the right button if the letter was a vowel and the left button if the letter was a consonant. In the *case discrimination* condition (task B), subject had to press the right button if the letter was upper case and the left button if the letter was lower case. In these two conditions, the color of the letter was irrelevant and changed pseudo-randomly. The control condition was composed of the mean of these two discrimination tasks [(A + B)/2] (i.e., the two single tasks averaged together).

In the *task switching* condition, subject had to perform one of the two tasks mentioned above according to the color of the letter, red letters indicating the vowel–consonant discrimination condition while green letters indicated the case discrimination condition. Switch trials were defined as trials in which subjects had to alternate from the previous task (from task A to task B or from Task B to task A). Repeat trials were defined as trials in which subjects repeated the same task (from task A to task A or from task B to task B). In the three conditions, the Inter Stimuli Interval (ISI) was 3 s and the stimulus duration was 800 ms. The letters used in the different conditions were taken among the following set of letters (i.e., upper or lower cases, red or green): c, d, f, h, k, m, p, r, t, v, a, e, i, o, u, y. All conditions were constructed by pseudo-randomly choosing among this set of letters that were equated for the number of vowels/consonants, upper/lower case letters, red/green letters and left/right responses. Each run was pseudo-randomly ordered according to a Latin square design, so that each condition appeared only once at different serial positions within a run and control and switch conditions alternated. The order of runs was also counterbalanced across subjects.

### fMRI Acquisition

Subjects were scanned twice, on the 39^th^ day of each treatment stage, i.e. on the 11^th^ day of the 2^nd^ and of the 4^th^ month (Fig. 1). High-resolution images were obtained with a 1.5 T Siemens Magnetom Sonata Maestro Class MRI System (Siemens, Munich, Germany). Two higher order shimming procedures were completed: the first covering the whole brain, whereas the second covered the orbitofrontal region. Five functional time series of 100 whole-brain images functional scans were performed with an EPI T2*-weighted sequence (TR/TE = 2500/60 ms, flip angle = 90°; FOV = 22 cm, acquisition matrix = 64 × 64, slices thickness = 4 mm). An axial anatomic T1-weighted sequence (TR/TE = 1970/3.93 (ms), inversion time TI = 1100 ms, FOV = 256 mm, acquisition matrix = 256 × 256, slice thickness = 1mm, number of slices = 26) along the anterior commissure/posterior commissure (i.e., AC/PC) line and covering the whole brain was also acquired. For hormonal measures and fMRI data analyses, see Supplemental methods.

## Additional Information

**How to cite this article:** Girard, R. *et al*. Hormone therapy at early post-menopause increases cognitive control-related prefrontal activity. *Sci. Rep.* **7**, 44917; doi: 10.1038/srep44917 (2017).

**Publisher's note:** Springer Nature remains neutral with regard to jurisdictional claims in published maps and institutional affiliations.

## Supplementary Material

Supplementary Information

## Figures and Tables

**Table 1:** Brains regions showing increased activity during task switching compared to control condition.

Main effect of the task switching > control condition				
Anatomical Structure	x	y	z	T value
(Broadmann’s area)				
**Frontal**				
Left Superior frontal gyrus (BA 6)	−21	−7	61	3.78
Right Superior frontal gyrus (BA 9)*	18	47	40	5.75
Left Middle frontal gyrus (BA 44)*	−45	20	37	11.66
Right Middle frontal gyrus (BA 45)*	51	29	34	7.43
Left Inferior frontal gyrus (BA 45)*	−45	35	4	11.66
Right Inferior frontal gyrus (BA 47)*	45	38	−8	7.60
Left Precentral gyrus (BA 6)	−27	−10	58	3.90
Left Putamen*	−27	−1	7	7.45
Left SMA (BA 6)	−3	−1	61	6.99
**Parietal**				
Left Superior parietal lobule (BA 7)*	−21	−64	58	6.16
Right Superior parietal lobule (BA 40)*	42	−55	55	7.09
Left Inferior parietal lobule (BA 7)*	−33	−55	49	9.65
Left Precuneus (BA 7)*	−12	−70	46	6.63
Right Angular gyrus (BA 7)*	36	−58	46	5.89
**Temporal**				
Left Middle temporal gyrus (BA 20)	−45	−22	−17	6.16
Right Inferior temporal gyrus (BA 37)*	60	−61	−8	5.04
Right Temporal pole (BA 38)*	54	11	−5	3.97
Right Fusiform gyrus (BA 20)	39	−22	−20	6.64
**Occipital**				
Left Superior occipital gyrus (BA 19)*	−18	−73	40	7.11
Left Middle occpital gyrus (BA 18)	−36	−91	−8	4.05
Left Inferior occipital gyrus (BA 19)*	−42	−79	−11	7.33
Right Inferior occipital gyrus (BA 19)	39	−88	−5	4.58
Left Calcarine gyrus (BA 19)*	−21	−73	7	5.70
Right Calcarine gyrus (BA 17)	21	−61	10	6.10
**Cerebellum**				
Left Anterior lobe (BA 37)*	−24	−43	−29	7.56
Right Anterior lobe (BA 37)*	18	−58	−26	8.43
Left Posterior lobe (BA 19)*	−30	−67	−23	6.17
Left Vermis*	−3	−46	7	6.93
Right Vermis*	0	−55	−8	6.14
**Cingulate cortex**				
Right Middle cingulate cortex (BA 23)	9	−31	34	5.27
**Insula**				
Right Insula (BA 47)*	42	23	−2	6.47

The coordinates are given within the framework standardized stereotaxic brain area atlas of Talairach and Tournoux (1988). All areas were significant at p < 0.001, uncorrected, *p < 0.05 FWE cluster-wise corrected.

**Table 2:** Brains regions showing increased activity after hormone therapy compared with placebo during task switching blocks relative to control.

Hormone therapy > Placebo				
Anatomical Structure (Broadmann’s area)	x	y	z	T-value
**Frontal**				
Right Middle frontal gyrus (BA 46)**	42	35	40	4.13
Left Inferior frontal gyrus (BA 48)	−36	23	10	4.19
Right Inferior frontal gyrus (BA 48)**	33	26	16	4.51
Left Paracentral lobule (BA 4)	−15	−28	67	3.49
Right Rolandic Operculum (insula)*	63	−4	10	4.52
**Cingulate**				
Left Anterior cingulate cortex (BA 24)**	0	14	28	3.70
Right Middle Cingulate gyrus (BA 24)	3	8	37	3.63
**Hippocampus**				
Left Hippocampus	−18	−25	−8	3.57
**Thalamus**				
Left Thalamus	−15	−16	10	4.31
**Temporal**				
Right Superior temporal gyrus (BA 48)	63	2	4	4.38
Right Middle temporal gyrus (BA 20)	63	−40	−11	3.50
Right Inferior temporal gyrus (BA 20)	57	−28	−20	4.18
Left Temporal Pole (BA 48)	−48	8	−8	4.81
Right Temporal Pole (BA 20)	39	11	−29	3.83
Right Heschls gyrus (BA 48)*	60	−7	7	4.48
**Midbrain**				
Right Vermis (BA 30)	6	−40	−17	3.57
**Insula**				
Left Insula (BA 48)	−39	5	−2	3.55

The coordinates are given within the framework standardized stereotaxic brain area atlas of Talairach and Tournoux (1988). All areas were significant at p < 0.001 uncorrected, *p < 0.05 FWE cluster-wise corrected, **p < 0.05 SVC FWE corrected.
