# Supplementary material for: Hormone therapy at early post-menopause increases cognitive control-related prefrontal activity
Source: Sci Rep. 2017 Mar 21;7:44917. doi: 10.1038/srep44917 (PMC5359606; doi:10.1038/srep44917)
Supplement: Supplementary Information [file srep44917-s1.pdf]

Supplementary informations

Hormone therapy at early post-menopause increases cognitive control-related prefrontal activity

Abbreviated title: Effect of Hormone Therapy on cognitive control

Romuald Girard [*], Elise Météreau [*], Julie Thomas [*], Michel Pugeat [†], Chen Qu[*°]

and Jean-Claude Dreher[*]

**Neuroeconomics, Reward and Decision-making Team, Institut des Sciences Cognitives Marc Jeannerod, Centre National de la Recherche Scientifique, 69675 Bron, France*

° Psychology department, South China Normal University, Guangzhou, China

[†] INSERM U 863, Steroid Hormones laboratory, 69765 Bron (Lyon), France.

Supplementary Methods

Hormonal measures

Four venous blood samples were drawn to measure estradiol plasma level: one before the study to check the perimenopausal status, one on the first day of inclusion in the study and the last two before each fMRI session. The samples were centrifuged, aliquoted, and stored at -70°C until time of assay. Serum 17β-estradiol was measured by tritiated radioimmunoassay after diethylether extraction. The anti-estradiol antibody was raised by immunization of rabbit with estradiol-6-CMO-BSA. The antibody cross-reacted by 8.8% with 6-OH-estradiol, 3.0% with 16-ceto-17β-estradiol, 1% with estrone, 0.15% with estriol, 0.4% with 17α-estradiol, 0.1% with ethynyl-estradiol and <0.001% with testosterone or 17α-OH-progesterone. The detection limit was 11 pmol/L and the inter-assay coefficient of variation was 12.1% for 21 pmol/L, 6.0% for 100 pmol/L and 9.4 % for 169 pmol/L. An ANOVA was performed with HT/placebo as independent factor to test the difference between estradiol levels on the two scanning days.

fMRI Data Analysis

The fMRI data from both scanning sessions (HT and placebo) were preprocessed and analyzed using the SPM8 software (Wellcome Department of Cognitive Neurology, London, UK), implemented in Matlab 7.7 (The MathWorks, Natick, USA).

The four initial scans were discarded to only use data acquired during steady state magnetization. First, images were corrected for spatially realigned to the first image from the first session using a six-parameter, rigid-body transformation, unwrapped to correct for geometric distortions. Inhomogeneities distortions-related correction maps were created with fieldmap SPM toolbox, using the phase of non-EPI gradient echo images measured at two echo times (5.19 ms for the first echo and 9.95 ms for the second). Using the Mazaika approach, sequences with significant scan-to-scan motion artifact greater than 0.5 mm/TR or a variation in global intensity greater than 1.3% were considered corrupted and corrected using the linear interpolation algorithm of the ArtRepair SPM toolbox [1]. The overall percentage of TRs removed for task switching and control condition was respectively 5.6% and 6.15% (overall 6%). No significant differences in the percentage of TRs removed were observed between whether task switching and control conditions or between treatment and placebo. For each participant, T1-weighted anatomical images were coregistered to the mean EPI, and segmented into white and gray matter. Then, grey matter was normalized using standard Montreal Neurological Institute (MNI) space template conforming to the Talairach orientation system [2] by applying a 12-parameter affine transformation followed by a nonlinear warping [3]. The computed transformation parameters were, then applied to all of the functional images, interpolated to a final voxel size of 3×3×3 mm^3. Finally, an equivalent spatial smoothing of 8 mm full width at half maximum (i.e., FWHM) Gaussian kernel was applied.

A random-effect, epoch-related statistical analysis was performed with a two-level procedure. At the single-subject level, statistical parametric maps were computed from fMRI time series as a block design using a separate general linear model for each subject and each session. A set of boxcar functions, modeling the duration of each

conditions (Task A, Task B and Task Switching) separately, was convolved with a synthetic hemodynamic response function lasting 78 seconds. The data were high-pass filtered (128 s cutoff) to remove low-frequency drifts and serial correlations were accounted for by an autoregressive model of the first order. Contrast images were calculated for each participant in both hormonal treatment sessions to compare brain activity during task switching relative to the control condition.

The first levels maps were then submitted to a second level analysis in which subjects were treated as a random effect, using a flexible factorial model with subjects, hormonal state (placebo, HT) and conditions (task switching and control) as factors. First, we investigated the resulting statistical maps for the comparison between task switching relative to control, regardless the hormonal state (i.e. task switching > Tasks (A+B)/2). Subsequently, we directly compared the two hormonal states (HT compared to placebo) during task switching relative to control, using a flexible factorial design with hormonal state, task switching and control conditions as factors. In all our comparisons, we used a threshold of $p<0.001$ uncorrected.

Activations localization and reported statistics

Coordinates tables are given within the framework standardized stereotaxic brain area atlas of Talairach and Tournoux [2] and list all the regions reaching a statistical significant level with a voxel-wise threshold of $p < 0.001$, uncorrected. Anatomic labeling of activated regions was performed using Anatomy and WFU PickAtlas SPM toolboxes. Moreover, a small volume correction (SVC) analysis was performed with a Family Wise Error (FWE) corrected significance threshold of $p<0.05$, in a 10-mm radius sphere centered on the dlPFC [x, y, z=42, 38, 28], ACC [x, y, z=4, 8, 48] and right ventro-lateral prefrontal cortex [x, y, z=30, 34, 15], based on coordinates taken from meta-analyses on task-switching [4,5].

Region Of Interest Analysis

Regions Of Interest (ROI) were used to determine mean beta weights for each condition in the two hormonal states. Regions of interest were defined functionally from the prefrontal activity observed when averaging the whole-brain analyses taken from two hormonal states and conducted with the extension of SPM MarsBaR (http://marsbar.sourceforge.net/). For HT compared to placebo during task switching relative to control, the functional clusters of interest were the dorsolateral prefrontal cortex and the inferior frontal junction/anterior insula bilaterally. For the anterior cingulate cortex, betas values were extracted on peak-voxel activity. In the statistical comparison between placebo and HT, the functional ROI was in the right anterior medial prefrontal cortex. Figures 5 and Supp. Mat 1 show the parameter estimates obtained from GLM1 in each ROI. Note that no statistical test was performed on these parameter estimates because the ROI analysis is not independent from the whole-brain analysis.

The ROI analyses in the left and right pre-SMA (**Figure S2**) were performed using anatomic ROIs independently created from the WFU PickAtlas SPM toolbox. The betas estimated were extracted from the task switching > control comparison and compare using a paired t-test (two-tails).

Supplementary Results

Assessment of women quality of life under HT and placebo

On the first day of the visit and at the end of each week of our 16 weeks study, women filled a simplified Women Health Questionnaire (WHQ) to assess 9 domains of symptom experience relevant to the menopause and associated with psychosocial factors, including depressive mood, somatic symptoms, memory/concentration, vasomotor symptoms, anxiety/fear, sexual behavior, sleep problems, menstrual symptoms and attractiveness. The scores obtained for the 9 domains assessed after the 8 weeks under HT and after the 8 weeks under placebo were averaged separately for each subject (Fig.2S). A paired t-test analysis did not show significantly difference between HT and placebo (all: $P > 0.05$), suggesting that larger cohort neuropsychological studies is needed to ensure sufficient power to observe differences between treatment types.

References

1 Mazaika, P. K., Hoeft, F., Glover, G. H. & Reiss, A. L. Methods and software for fMRI analysis of clinical subjects. *Neuroimage* **47**, S58 (2009).

2 Tailarach, J. & Tournoux, P. *Co-planar stereotaxic atlas of the human brain: 3-dimensional proportional system—an approach to cerebral imaging*. (Thieme Medical Publishers, 1988).

3 Ashburner, J. & Friston, K. J. Nonlinear spatial normalization using basis functions. *Hum Brain Mapp* **7**, 254-266 (1999).

4 Derrfuss, J., Brass, M., Neumann, J. & von Cramon, D. Y. Involvement of the inferior frontal junction in cognitive control: meta-analyses of switching and Stroop studies. *Hum Brain Mapp* **25**, 22-34, doi:10.1002/hbm.20127 (2005).

5 Wager, T. D., Jonides, J. & Reading, S. Neuroimaging studies of shifting attention: a meta-analysis. *Neuroimage* **22**, 1679-1693, doi:10.1016/j.neuroimage.2004.03.052 (2004).

Supplementary Figures

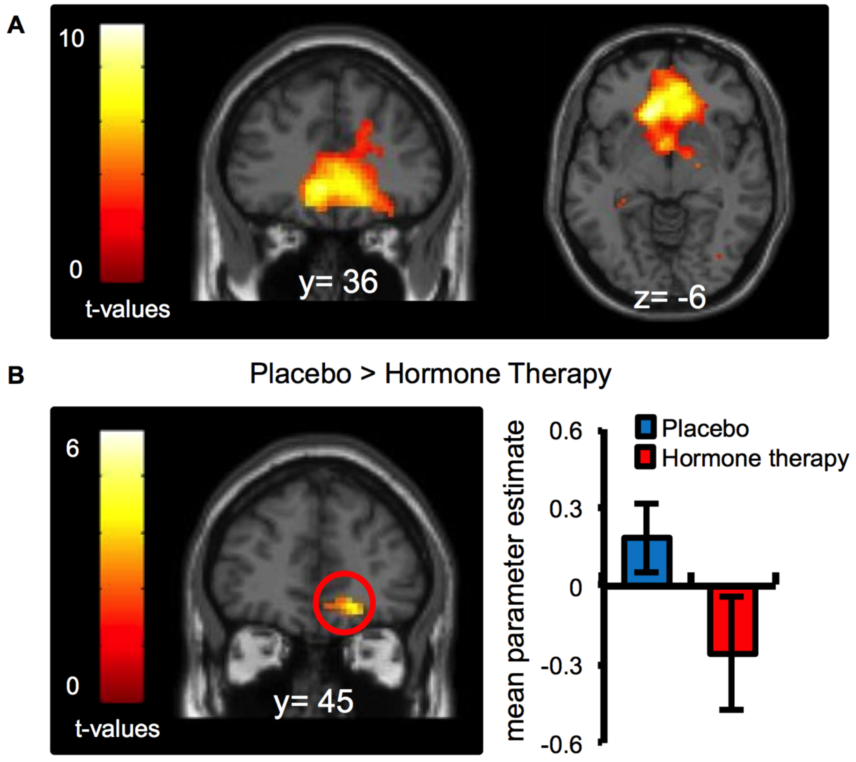

Figure S1. Anterior medial prefrontal cortex engaged by the comparison 'control >task switching', regardless of treatment type. (A) In the comparison 'control > task switching', a relative deactivation was observed in a large anterior medial frontal cortex region (Display threshold: voxel-wise $p<0.001$, uncorrected; cluster-wise $p<0.05$, FWE corrected). (B) Direct statistical comparison, between Placebo and HT in 'control> task switching', showed higher BOLD responses in the right anterior medial prefrontal cortex (Display threshold, $p<0.005$, uncorrected). The activation maps are overlaid on coronal sections of a structural template MRI. The color bar indicates t values.

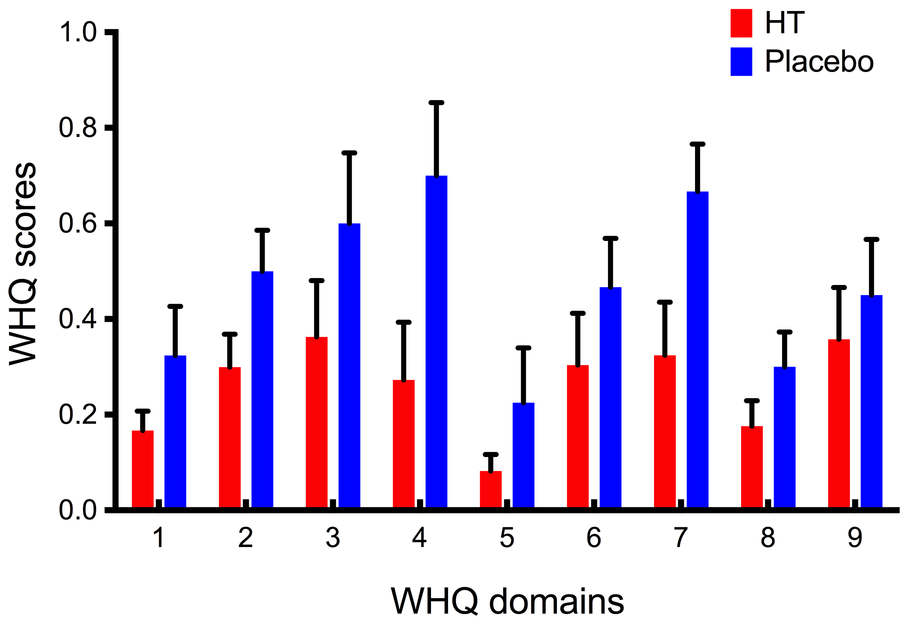

Figure S2. Evaluation of women's quality of life after 8 weeks under placebo and after 8 weeks after HT. Scores of the 9 assessed domains ((1) depressed mood, (2) somatic symptoms, (3) memory/concentration, (4) vasomotor symptoms, (5) anxiety/fears, (6) sexual behavior, (7) sleep problems, (8) menstrual symptoms, (9) attractiveness) of the Women's Health Quality of life questionnaires assessed all along the placebo and HT period reported for all subjects after averaging their individual scores.”

Table S1. Estradiol level in pmol/L (running order) after placebo and hormone therapy.

	Placebo	Hormone therapy
Subject 1	19 (day 1)	236
Subject 2	57 (day 1)	200
Subject 3	19	82 (day 1)
Subject 4	19	205 (day1)
Subject 5	19 (day 1)	228
Subject 6	19 (day 1)	104
Subject 7	27	28 (day 1)
Subject 8	21	212 (day 1)
Subject 9	19	109 (day 1)
Subject 10	26	74 (day 1)
Subject 11	19 (day 1)	102
Subject 12	19 (day 1)	132

Table S2. Brains regions showing increased activity after placebo compared with hormone therapy during task switching blocks relative to control.

Placebo > Hormone therapy

Anatomical Structure (Broadmann's area)	x	y	z	T value
**Frontal**				
Left Superior frontal gyrus (BA 8)	-18	20	46	4.85
Right Superior frontal gyrus (BA 10)	18	56	22	4.84
Left Middle frontal gyrus (BA 6)	-27	8	49	3.64
Right Middle frontal gyrus (BA 11)	21	44	-11	4.83
Left Inferior frontal gyrus (BA 45)	-42	44	4	4.85
Right Inferior frontal gyrus (BA 45)	54	26	25	4.36
Left Superior Medial frontal gyrus (BA 32)	-12	35	37	4.53
Right Superior Medial frontal gyrus (BA 10)	9	62	19	3.39
Left Paracentral lobule	-9	-34	55	3.4
Right Paracentral lobule (BA 4) *	12	-31	64	4.25
Right Precentral gyrus (BA 4)	36	-19	49	3.87
Right Putamen (BA 47) *	24	23	-5	4.8
Left SMA (BA 6)	-12	2	64	3.5
**Parietal**				
Left Superior parietal lobule (BA 5) *	-18	-55	61	6.02
Right Superior parietal lobule (BA 5) *	21	-52	58	5
Left Inferior parietal lobule (BA 2)	-54	-31	37	3.86
Left Postcentral gyrus (BA 5) *	-15	-46	70	5.16
Right Postcentral gyrus (BA 3)	48	-22	40	3.99
Left Supramarginal gyrus (BA 2)	-60	-28	37	3.81
Precuneus *	-6	-64	28	4.67
Left Angular (BA 22) *	-57	-58	25	4.26
Right Angular (BA 40)	54	-49	28	3.41
**Occipital**				
Left Superior occipital gyrus (BA 19)	-18	-82	22	3.68
Right Superior occipital gyrus (BA 18) *	24	-88	16	5.65
Left Middle occipital gyrus (BA 18)	-21	-97	-2	4.11
Right Middle occipital gyrus (BA 19) *	30	-67	4	6.51
Right Inferior occipital gyrus (BA 19) *	51	-76	-2	4.93
Right Calcarine gyrus (BA 18) *	24	-97	1	4.84
Left Cuneus (BA 19)	-12	-82	31	3.94
Right Lingual gyrus (BA 18)	6	-64	4	3.56
**Temporal**				
Right Superior Temporal Gyrus (BA 22)	57	-16	-5	3.79
Left Middle temporal gyrus (BA 21) *	-45	-52	16	5.34
Right Middle temporal gyrus (BA 21) *	60	2	-20	6.21
Right Inferior temporal gyrus (BA 37) *	51	-55	-14	5.04
Right Fusiform (BA 19) *	36	-70	-20	5.72
**Cerebellum**				
Right Anterior lobe (BA 30) *	18	-37	-23	4.89
Left Posterior lobe (BA 19) *	-6	-76	-32	5.42
Right Posterior lobe *	21	-76	-32	5.25
Right Vermis *	6	-49	1	4.46
**Cingulate cortex**				
Left Posterior cingulate (BA 23) *	-6	-49	22	7.02
Left Middle cingulate cortex	-12	-40	52	3.74
**Hippocampus**				
Left Hippocampus(BA 35)	-18	-13	-14	3.54
Right Hippocampus (BA 37) *	24	-40	4	4.78
**Thalamus**				
Right Thalamus *	9	-19	19	5.85
**Insula**				
Right Insula (BA 47) *	33	20	-14	3.7
**Pallidum**				
Left Pallidum	-12	5	-2	4.58
**Caudate**				
Right Caudate nucleus (BA 25)	9	8	-8	3.63

*p<0.05 FWE cluster-wise corrected
